# A Social Ecological Model of Vulnerability and Resilience in Older Adults During the Covid-19 Pandemic

**DOI:** 10.1093/geroni/igab046.505

**Published:** 2021-12-17

**Authors:** Carolyn Aldwin, Heidi Igarashi, Maria Kurth, Hye Soo Lee, Soyoung Choun, Dylan Lee

**Affiliations:** 1 Oregon State University, Corvallis, Oregon, United States; 2 Oregon State University, Oregon State University, Oregon, United States; 3 Oregon State University, Wilsonville, Oregon, United States

## Abstract

Objectives: We used a social ecological model to examine vulnerability and resilience among older adults during the COVID-19 pandemic. Methods: We analyzed two open-ended questions included in a survey of 235 respondents, 51–95 years old (M = 71.35; SD = 7.39; 74% female), which asked about COVID-19-related difficulties and positive experiences during the past week. We identified three different levels for difficulties and positives at the personal, interpersonal, and/or societal levels. Results: Fewer than half of the respondents reported on difficulties (41%) and positives (40%) just at the personal level. In terms of crossing levels, people were most likely to report events spanning the personal and interpersonal levels (14% and 18%, respectively). A few individuals reported difficulties and positives at the societal level. Discussion: Older individuals were acutely aware of challenges and positives existing at all three levels, and contributed to resources at the interpersonal and community levels.

